# Inhibitor of DNA-Binding/Differentiation Proteins and Environmental Toxicants: Genomic Impact on the Onset of Depressive Dysfunction

**DOI:** 10.3390/medsci7010007

**Published:** 2019-01-09

**Authors:** Vincent Avecilla, Andrea Avecilla

**Affiliations:** 1 Department of Environmental Health Sciences, Robert Stempel College of Public Health & Social Work, Florida International University, Miami, FL 33199, USA; 2 Celgene Corporation, Summit, NJ 07901, USA; 3 Department of Clinical Psychology, University of Massachusetts Dartmouth, North Dartmouth, MA 02747, USA; aavecilla@umassd.edu

**Keywords:** depression, estrogenic endocrine disruptor, environmental factor, inhibitor of differentiation, mental disorder

## Abstract

The ongoing growth of the international occurrence of depression and its ability to co-occur with other serious medical disorders, such as heart disease, cancer, diabetes, and Parkinson’s disease, is a current public health problem. Inhibitor of DNA-Binding/Differentiation (ID) proteins are part of a group of transcriptional factors that have shown involvement in neurocognitive disorders and, therefore, may have influence on depressive disorders. Previously, it has been established that exposure to environmental estrogenic endocrine disruptors (EEDs), such as polychlorinated biphenyls (PCBs) and bisphenol A (BPA), have played an important role in the modulation of depressive disorders. Hence, based on many studies, we consider the impact of these environmental pollutants on the group of ID proteins and how they impact depressive outcomes. Improved knowledge of how ID proteins interact with depressive disorders, through EED exposure, will contribute essential evidence that can further benefit our public health community with innovative knowledge to prevent these types of mental illnesses.

## 1. Introduction

Depression is a shared but serious mood disorder. It can cause severe symptoms that affect how you think, feel, and handle daily activities, such as working, eating, or sleeping. Depression is one of the most common mental disorders in the United States [1,2]. An estimated 16.2 million adults in the United States have, at minimum, one depressive episode, which signifies 6.7% of all U.S. adults. Furthermore, depressive episodes are greater among adult females (8.5%), when compared to males (4.8%) [1,2]. Depression can occur at any age but often starts in adulthood. There are numerous forms of depression, which may cultivate under distinctive conditions, such as the persistent depressive disorder, psychotic depression, postpartum depression, bipolar disorder, and seasonal affective disorder [1,2]. Today, there are many factors that can onset the development of depression. Currently, there is a prerequisite to identify how environmental pollutants, such as estrogenic endocrine disruptors (EEDs), contribute to the depressive disorder predisposition.

Estrogen has been previously established to have frequent purposes, including the regulation of endocrine expansion and growth, alongside metabolism [3]. Additionally, estrogen has been seen to affect depressive outcomes [4,5,6]. As a result of this, depression may be predisposed to EED exposure. These categories of pollutants have the capability to alter hormone production or function. This group includes phytoestrogens, heavy metals, and anthropogenic chemicals, such as polychlorinated biphenyls (PCBs), bisphenol A (BPA), arsenic, phthalates, and diethylstilbestrol (DES) [7,8,9,10,11]. Findings have demonstrated connections between EED exposure and depression [12,13,14,15,16,17]. Based on discoveries that demonstrate that the Inhibitor of DNA-Binding/Differentiation (ID) proteins have been connected with depression [18,19,20,21,22], we will also emphasize how environmental EED exposure may modulate the depression outcomes via ID proteins. The goal of this review is to make links between ID proteins to EED interactions, thus, leading to altered results in depression. Additional research in these competences may reveal novel or more valuable modalities and aid to deliver methodologies for prevention of this disorder.

## 2. Inhibitor of DNA Binding/Differentiation

### 2.1. Background

Inhibitor of DNA-Binding/Differentiation proteins are encoded by four genes: *ID1*, *ID2*, *ID3*, and *ID4*, respectively that create a group of transcriptional regulators [23,24]. These groups of transcriptional factors, belonging to the basic helix-loop-helix family (E proteins: E12, E47), which are regulated by ID proteins [25,26], are involved in the modulation of biological processes, such as the cell cycle control, angiogenesis or apoptosis, cell differentiation and proliferation, metastasis, and senescence [27,28]. Inhibitor of DNA-Binding/Differentiation proteins play an essential role in the nervous tissue biology and remain constant through the nervous tissue development [29,30,31]. Depression may co-exist with other neurocognitive disorders where nervous tissue development is an important factor, such as Parkinson’s disease, dementia, and Alzheimer’s [32,33]. Reactive oxygen species (ROS) have demonstrated to induce ID protein-facilitated dysregulation and cell proliferation in both in vivo and in vitro settings [34,35,36]. Inhibitor of DNA-Binding/Differentiation proteins, such as ID3, are redox-sensitive; it has been shown to be an essential factor of the ROS-stimulated proliferation of endothelial cells and estradiol (E2) to PCB153 [37,38,39]. Depression is triggered when neurotransmitters, that are chemical messengers that help the brain communicate with parts of the body, are out of equilibrium. Low levels of neurotransmitters may play a role in why some individuals are more predisposed to depression, including dopamine, norepinephrine, and serotonin [1]. It has been shown that these neurotransmitters have been interconnected with various levels of ROS [40,41,42,43]. Modeling of the mitochondria has been used to explain the biphasic nature of how respiration, in both depression and bipolar disorder, transition between the varying levels of ROS [40]. Additionally, it has also been determined that an increase in anxiety and depression might trigger an increase in norepinephrine, causing ROS formation, therefore, increasing the risk of high blood pressure and cardiovascular disease in hypertensive individuals [41]. As levels of ROS increase, human tissue becomes affected, at a molecular level, over a duration of time. Since ID proteins are demonstrated to be redox-sensitive, we predict environmental toxicants, such as EEDs, may enhance the ROS-stimulated levels of ID proteins, thus, causing the onset of depressive dysfunction.

### 2.2. Inhibitor of DNA Binding/Differentiation and Depressive Disorders

There has been evidence demonstrating the role of ID proteins in depressive disorders. Disruptions in behavioral and circadian rhythm-connected physiological processes are regularly seen in depressed patients. Nonetheless, contribution of the circadian system in depressive pathophysiology is incompletely comprehended. Savalli et al. demonstrated that stress-stimulated anhedonic behavior in mice is connected with agitated diurnal oscillation of the expression of the genes *Rev-erbα*, *ROR*-*β*, *ROR*-*γ*, *CRY2*, *PER1*, *CLOCK*, and *ID2* in the mouse basolateral amygdala. The aberrant control of the diurnal rhythmicity connected to depression may directly result from the mental illness itself, and thus, establish an animal model for additional exploration [20].

Epigenetic markers were previously used to determine various rating of depression in maltreated children. Weder et al. performed a genome-wide methylation study in 94 maltreated and 96 healthy non-traumatized children, with saliva-derived DNA. Results showed that methylation in three genes were considered significant predictors of depression, including Tubulin Polymerization Promoting Protein (*TPPP*), DNA-Binding Protein Inhibitor-3 (*ID3*), and Glutamate *N*-Methyl-d-Aspartic Acid Receptor (*GRIN1*). These are biologically applicable, with the *TPPP* being involved in the neural circuitry growth, *ID3* being involved in stress-response, and *GRIN1* being involved in neural pliability, suggesting that epigenetic changes in these genes, particularly with the combination of maltreatment, might present a risk for depression in children [21].

Furthermore, Motalvo-Ortiz et al. validated the epigenetic changes of the genes *GRIN1*, *ID3*, and *TPPP*. Secondary analysis was conducted using the gene expression data obtained from the medial prefrontal cortex (mPFC) tissue of mice that underwent a model of maternal neglect, including early weaning (MSEW) and maternal separation. Depression-like phenotype data from the elevated plus maze (EPM) and forced swimming tests (FST), were also available. Results revealed the gene expression of *ID3*, *TPPP*, and *GRIN1* in the mPFC to indicate behavioral alterations in the FST and EPM, thus, further supporting the role of these genes in the depressive phenotypes, following early life stress [22].

### 2.3. Inhibitor of DNA Binding/Differentiation and Environmental Pollutants

The contribution of ID3 to multifaceted ailments, via metabolic distresses and environmental influences has previously been determined [18]. Inhibitor of DNA-Binding/Differentiation 3 also influences metabolic health and obesity, in response to environmental stressors [44]. Inhibitor of DNA-Binding/Differentiation proteins have been seen linked to various types of EEDs, such as PCBs, BPA, arsenic, and phthalates. Mechanisms responsible for initiating microvascular damage continue to be inadequately definite, while aspects such as oxidative stress induced by environmental toxicants, have been suggested. Association in the development of proliferative vascular lesions, via increased neovascularization, has been brought to attention. The existing data have demonstrated how ROS might contribute to the neo-vascular phenotype progression, via the PCBs, with the objective of demonstrating the role of the environmental toxicants in endothelial dysfunction, with a focus on *ID3*. Polychlorinated biphenyl-stimulated ROS-intermediated neo-vascular phenotype, furthermore, depend on Pyk2 (Protein-tyrosine kinase 2) and *ID3*. Additionally, PCB153 treatment expanded the endothelial spheroids’ measurement with conditions that work on behalf of the stem cell spheroid clonal selection. Higher ID3 protein expression, matched with a greater quantity of oxidative DNA injury marker 8-OHdG, in blood vessels. Overall, this shows the conceivable function of *ID3* in regulating the micro-vascular lesion growth and vascular endothelial cell survival, driven by environmental toxicants, such as PCB153 [37,38]. Another study investigated the process through which an exposure to BPA stimulated reproductive anomalies in the adult male testis. Adult C57/BI6 males were exposed to sesame oil, BPA, or diethylstilbestrol (DES), as a positive control from gestational days 10 to 16, and were observed. Adult mRNA levels of genes associated with sexual maturation and differentiation, *ID2* and *GATA4*, were lower only in the testes exposed to DES. At the molecular level, DES exposure via in utero, not BPA, led to decreased mRNA gene expression, connected with Sertoli cell differentiation [45].

Arsenic has also been seen to be involved with ID proteins. Arsenic exposure is known to be a risk factor for various cancers. Tsai et al. aimed to investigate the contribution of *ID1* and connected signaling molecules in the arsenic-mediated angiogenesis. The initial screening led to low arsenic contractions, showing cellular responses, including angiogenic activity and enhanced endothelial cell viability, alongside increased *ID1* expression. Stimulated arsenic angiogenesis was suppressed in the ID1-knocked down cells, compared to the control cells. Additionally, angiogenic action and arsenic-stimulated expression of *ID1* showed mediation by PI3K/Akt, nitric oxide synthase (NOS), and nuclear factor κ B (NF-κB) signaling. As a result, the data showed that *ID1* regulated angiogenesis supported by arsenic, and *ID1* may be an anti-angiogenesis target for cancer associated with arsenic [46]. Furthermore, it was found that treatment with stress-stimulated metalloid arsenite, a chemical compound containing an arsenic oxoanion, led to the accumulation of GFP-tagged *ID3* in the cytoplasm. Spaced N-terminal cysteine residues of *ID3* interacted with arsenic derivate phenylarsine oxide (PAO) and showed importance for the arsenite-produced cytoplasmic accumulation, which suggests that arsenite induces Chromosomal Maintenance 1 (CRM1) dependent nuclear export of *ID3*, by binding to the N-terminal cysteines. Overall, this indicates that *ID3* may be involved in the biological activities of arsenite [47].

Arsenic trioxide (ATO), an important oxide of arsenic is a main precursor to the other arsenic compounds. It has shown to strongly induce differentiation and apoptosis in acute promyleocytic leukemia, alongside cell cycle arrest in most solid tumors. Zhang et al. screened signaling pathways that are involved in antitumor mechanisms and molecules that contribute in the antitumor effects of ATO. Results demonstrated that after verification at the transcriptional and translational levels in four different cancer cells, *ID2* was identified as an ATO anti-tumor-connected protein. Furthermore, silencing of the *ID2* may enhance the ATO-stimulated cell proliferation inhibition in cancer cells [48]. Phthalates, which are also considered EEDs, are widely used in the production of plastic products and other consumer goods. In a study done by Yao et al., mono-(2-ethylhexyl) phthalate (MEHP) stimulates matrix metalloproteinase 2 (MMP2) expression in testicular embryonal carcinoma NT2/D1 cells, however, it has no important effect on the MMP9 expression. Additionally, MEHP treatment caused certain genes, including *GJA1* (Gap junction protein-α 1), *VCL* (vinculin), and *ID1* (inhibitor of DNA-binding protein-1) to down-regulate, while *CLDN6* (claudin-6) and *CTNNB1* (β 1-catenin) were up-regulated. Yao et al. provided insights into mechanisms that may account for modulating the progression of cancer, following exposure to phthalates [49].

## 3. Relationship between Environmental Toxicants and Depressive Disorders

Estrogenic endocrine disruptor exposure has been previously demonstrated in various animal and population studies with a focus on depression. Polychlorinated biphenyls have been connected with depressive symptoms. Data was collected from 178 individuals at two measurement time points. Polychlorinated biphenyls were analyzed in plasma through human bio-monitoring and depressive symptoms were validated via a questionnaire. Results demonstrated noteworthy mediation, over time, for dioxin-like, higher-chlorinated, and lower-chlorinated PCBs. Positive connections between the PCB exposures with depressive symptom severity was facilitated by the main dopamine (DA) metabolite homovanillic acid (HVA). Higher exposure was also linked with PCBs with lower concentration in urinary HVA. Overall, this indicates that links between PCB exposure and higher depressive symptoms, after one year, is mediated by the DA metabolite HVA as a substitute for the DA, which can help elucidate the principal neurochemical mechanisms of PCB-related depressive symptoms [50]. Additionally, studies suggest that exposure to BPA might contribute to neurobehavioral problems in childhood, which results in symptoms of anxiety and depression. Perera et al. investigated the association of prenatal BPA, observing sex-focused differences in both depressive and anxiety indications in children aged 10–12 years old. Important positive connections between symptoms of depression and anxiety and prenatal BPA were observed among boys but not girls aged 10–12 years old [51]. Similarly, BPA has also been addressed in animal studies investigating whether paternal BPA can affect emotions of male rats and their respected offspring. Eighteen adult rats (F0) received a BPA diet for 21 weeks and then mated with non-exposed females to produce offspring (F1). Behaviors were evaluated in various tests, including forced swimming test, elevated plus maze, and open-field test. Furthermore, their serum corticosterone was observed. Exposure to BPA stimulated higher anxiety behaviors in the F0 rats. Paternal exposure led to higher anxiety behaviors in the F1 females and aggravated depression behaviors in both sexes of F1 rats. This data suggests preconception-paternal-exposure to low-dose BPA might stimulate transgenerational sex-focused deficiencies in adult rats [51].

There also has been a relationship between arsenic and depression. A sample of 223 women was previously gathered from five public services in Chile. Data associated to arsenic exposure and urine samples for inorganic arsenic assessments were collected during the women’s second trimester pregnancy. Results revealed that the depression history, physical perception, number of children, age, and stressful maternity were associated with the postpartum score. Furthermore, the score was also associated with inorganic arsenic in women older than 25 years old [52]. Additionally, evidence indicates that sub-chronic exposure to arsenic causes cerebral neurodegeneration, which leads to disturbances associated with psychiatric disorders, such as depression. Chang et al. assessed the effects of sub-chronic arsenic exposure on the depression- and anxiety-like behaviors in both normal mice and a chemically-stimulated mouse model of depression via reserpine pretreatment. Results showed that arsenic exposure for four weeks increased anxiety-like behaviors in an elevated plus maze and an open field test, in normal mice, and eight weeks of exposure increased the depression-like behaviors in forced swimming test and tail suspension test, in reserpine pretreated mice. This reveals how sub-chronic exposure to arsenic induces anxiety-like behavior, while increasing the depression-like behavior in the mouse model of depression [53].

## 4. Genomic Interactions between the Inhibitor of DNA Binding/Differentiation, Environmental Toxicants, and Depression

**Figure 1:**
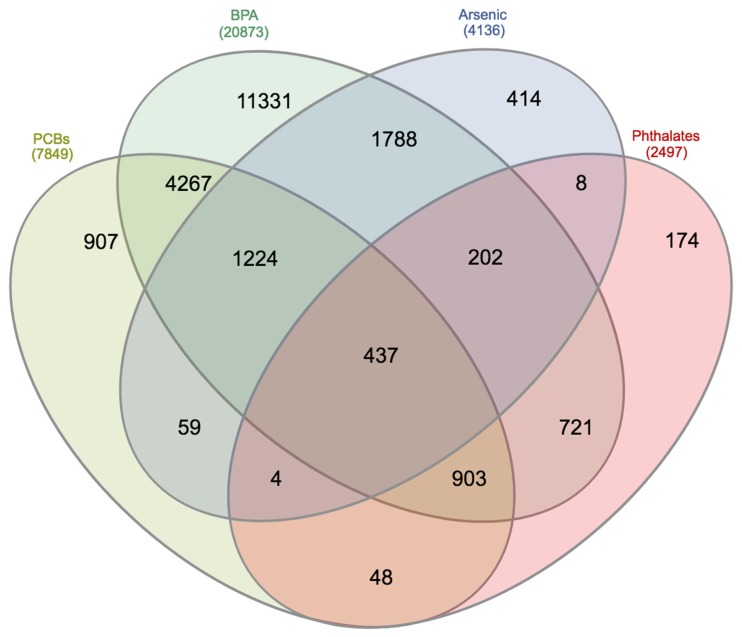
Venn diagram showing the interacting genes between estrogenic endocrine disruptors (EEDs): Polychlorinated biphenyls (PCBs; 7849 genes), Bisphenol A (BPA; 20873 genes), Arsenic (4136 genes), and Phthalates (2497 genes). Results show 437 overlapping genes.

**Figure 2:**
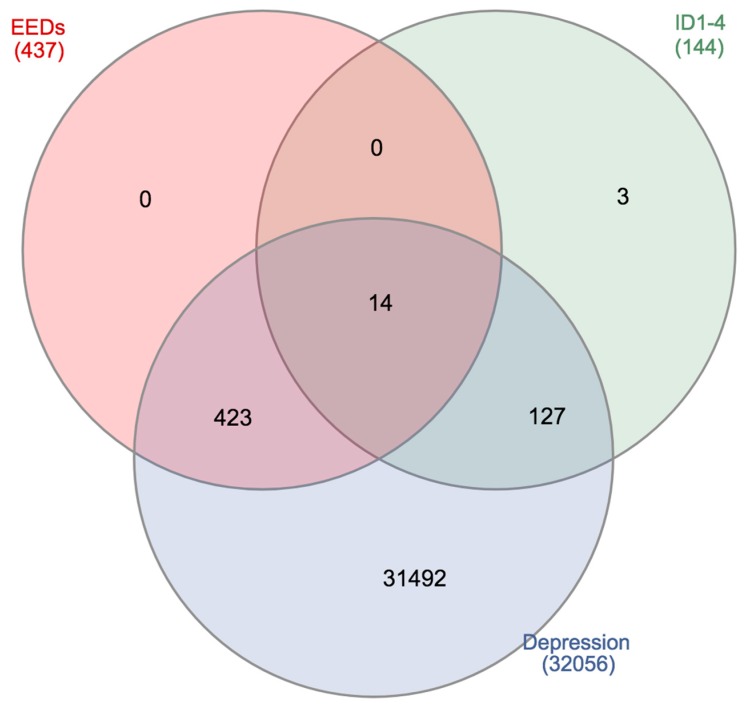
Venn diagram demonstrating the interacting genes between the overlapping estrogenic endocrine disruptors (EEDs; 437 genes), Inhibitor of DNA-Binding/Differentiation (ID) proteins (144 genes), and depression (32056 genes). Results reveal 14 overlapping genes.

**Figure 3:**
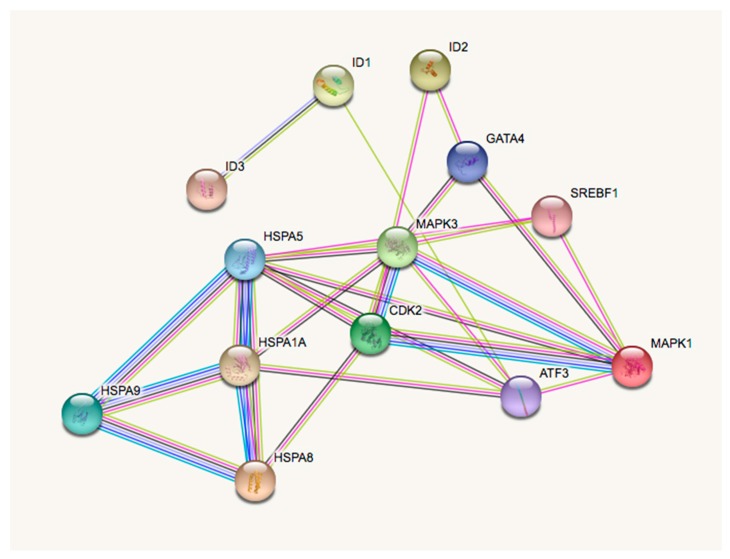
The gene network demonstrates a fully connected structure between the overlapping ID protein, EED, and depression genes.

To justify how the combination of ID proteins and environmental toxicants might contribute to depressive dysfunction at genomic levels, we integrated various publicly accessible tools, in order to help enhance our general understanding. We first used Comparative Toxicogenomic Database (CTD) [54] to support our understanding of gene interactions with ID proteins, various EEDs (including, PCBs, BPA, arsenic, and phthalates), and depressive disorders. Genes were curated for each of the categories, resulting in a common gene list, using a Venn diagram [55]. Figure 1 and Figure 2 show the interacting genes between the ID proteins, EEDs, and depression disorders. The results show the 437 interacting genes, overlapping among the EEDs, and the 14 interacting genes between ID proteins, EEDs, and depression. Overlapping gene results are, furthermore, shown in Table 1. To see how significant these genes are, we used the Kyoto Encyclopedia of Genes and Genomes Pathway to represent their genomic relation. We established that these 14 genes are represented in 33 molecular pathways [56]. The top 3 pathways are represented in Table 2 and it is revealed that each of these pathways have a role in depression and related ailments [57,58,59,60,61]. To validate that these 14 genes do interact and create a network, the STRING database was used to help provide protein-to-protein interaction [62,63]. As demonstrated in Figure 3, STRING delivers supplementary evidence that these genes create a genomic network, thus, elucidating the role of ID proteins and EED exposure on depression, via the associated interacting genes.

## 5. Conclusions

The Inhibitor of DNA-Binding/Differentiation proteins have been demonstrated to be connected with depression. Various studies have reported associations between depression and EED exposure, such as PCBs, BPA, arsenic, and phthalates. Based on evidence discussed in this review, we have shown that EED exposure may contribute to ID protein activation, in order to modify molecular mechanisms, thus, altering depressive dysfunction outcomes. Due to limited evidence caused by the novelty of this topic, it is essential to discuss the limitations of this study by conducting further research to assess how an exposure to EEDs and ID proteins might play a function in depressive perturbations. Results from this study would be beneficial in directing various public health and neurological professionals towards uncovering innovative opportunities that could potentially be used for prevention and treatment of these disorders, and beyond.

## Figures and Tables

**Table 1:** The 14 overlapping, interacting EED–ID protein-depression genes.

Gene Symbol	Gene Name
*ATF3*	Activating transcription factor 3
*CDK2*	Cyclin-dependent kinase 2
*ELOC*	Elongin C
*GATA4*	GATA binding protein 4
*HSPA1A*	Heat shock protein family A (Hsp70) member 1A
*HSPA5*	Heat shock protein family A (Hsp70) member 5
*HSPA8*	Heat shock protein family A (Hsp70) member 8
*HSPA9*	Heat shock protein family A (Hsp70) member 9
*ID1*	Inhibitor of DNA binding 1, HLH protein
*ID2*	Inhibitor of DNA binding 2, HLH protein
*ID3*	Inhibitor of DNA binding 3, HLH protein
*MAPK1*	Mitogen-activated protein kinase 1
*MAPK3*	Mitogen-activated protein kinase 3
*SREBF1*	Sterol regulatory element binding transcription factor 1

**Table 2:** The top three pathways with the 14 common overlapping genes with EEDs, ID proteins, and depression.

Pathway Name	Gene Count	*p*-Value	Genes
TGF-beta signaling pathway	5	9.34 × 10^−6^	*MAPK1, ID2, ID1, MAPK3, ID3*
Signaling pathways regulating pluripotency of stem cells	5	7.04 × 10^−5^	*MAPK1, ID2, ID1, MAPK3, ID3*
Estrogen signaling pathway	4	5.71 × 10^−4^	*MAPK1, MAPK3, HSPA1A, HSPA8*
